# A multimorbidity model for estimating health outcomes from the syndemic of injection drug use and associated infections in the United States

**DOI:** 10.1186/s12913-023-09773-1

**Published:** 2023-07-17

**Authors:** John J. Chiosi, Peter P. Mueller, Jagpreet Chhatwal, Andrea L. Ciaranello

**Affiliations:** 1 Medical Practice Evaluation Center and Division of Infectious Diseases, Massachusetts General Hospital, Boston, MA USA; 2 Harvard Medical School, Boston, MA USA; 3 Institute for Technology Assessment, Massachusetts General Hospital, Boston, MA USA

**Keywords:** People who inject drugs, Injection drug use, Opioid use disorder, HIV, Hepatitis C, Bacterial Infections, Endocarditis, Serious injection related infections, Drug overdose, Syndemic

## Abstract

**Background:**

Fatal drug overdoses and serious injection-related infections are rising in the US. Multiple concurrent infections in people who inject drugs (PWID) exacerbate poor health outcomes, but little is known about how the synergy among infections compounds clinical outcomes and costs. Injection drug use (IDU) converges multiple epidemics into a syndemic in the US, including opioid use and HIV. Estimated rates of new injection-related infections in the US are limited due to widely varying estimates of the number of PWID in the US, and in the absence of clinical trials and nationally representative longitudinal observational studies of PWID, simulation models provide important insights to policymakers for informed decisions.

**Methods:**

We developed and validated a MultimorbiditY model to Reduce Infections Associated with Drug use (MYRIAD). This microsimulation model of drug use and associated infections (HIV, hepatitis C virus [HCV], and severe bacterial infections) uses inputs derived from published data to estimate national level trends in the US. We used Latin hypercube sampling to calibrate model output against published data from 2015 to 2019 for fatal opioid overdose rates. We internally validated the model for HIV and HCV incidence and bacterial infection hospitalization rates among PWID. We identified best fitting parameter sets that met pre-established goodness-of-fit targets using the Pearson’s chi-square test. We externally validated the model by comparing model output to published fatal opioid overdose rates from 2020.

**Results:**

Out of 100 sample parameter sets for opioid use, the model produced 3 sets with well-fitting results to key calibration targets for fatal opioid overdose rates with Pearson’s chi-square test ranging from 1.56E-5 to 2.65E-5, and 2 sets that met validation targets. The model produced well-fitting results within validation targets for HIV and HCV incidence and serious bacterial infection hospitalization rates. From 2015 to 2019, the model estimated 120,000 injection-related overdose deaths, 17,000 new HIV infections, and 144,000 new HCV infections among PWID.

**Conclusions:**

This multimorbidity microsimulation model, populated with data from national surveillance data and published literature, accurately replicated fatal opioid overdose, incidence of HIV and HCV, and serious bacterial infections hospitalization rates. The MYRIAD model of IDU could be an important tool to assess clinical and economic outcomes related to IDU behavior and infections with serious morbidity and mortality for PWID.

**Supplementary Information:**

The online version contains supplementary material available at 10.1186/s12913-023-09773-1.

## Background

People who inject drugs (PWID) experience many complications from the substances used and from injection drug use (IDU). These complications can often occur together in PWID and include substance use disorder (e.g., opioid use disorder [OUD]), drug overdoses, and serious injection-related infections such as HIV, hepatitis C virus (HCV), and severe bacterial infections like endocarditis. These complications also escalate morbidity and mortality among PWID, particularly for those without access to harm reduction measures such as sterile syringes and other equipment. The increasing use of synthetic opioids, such as fentanyl, are driving fatal drug overdoses in the US [1]. PWID experience higher infection rates and poorer outcomes from infections (HIV, HCV, bacterial infections), creating a syndemic between drug use and these infections [2–5]. PWID may also experience infections concurrently (e.g., HIV/HCV co-infection), and one infection can exacerbate poor outcomes for another. For example, people living with HIV have an increased risk of both HCV-related fibrosis and cirrhosis as well as invasive bacterial infections such as endocarditis, particularly at lower CD4 counts [6, 7].

Despite high morbidity and mortality rates, nationally representative data from observational cohort studies on HIV, HCV, and bacterial infections among PWID and randomized control trials on interventions to reduce these infections among PWID are lacking. Policymakers often need information on long-term outcomes and cost-effective interventions to make important health decisions. In such cases, decision-analytic models can enrich the evaluation of clinical and public health interventions beyond the time horizons of clinical trials and observational studies. This importance is clear when studies focusing on PWID are limited, when trials have excluded PWID, or when important outcomes of public health interventions occur among people not engaged in care and thus are missed in trials and cohort studies [8]. However, previously published models of IDU do not consider multiple infections that PWID often experience concurrently nor account for the compounding clinical and economic effects of co-infections [9–12]. A multimorbidity model that accounts for multiple drug- and infection-related complications in PWID would improve estimations of the clinical and economic impact of the syndemic of drug use and related infections as well as more clearly demonstrate how interventions could impact multiple complications simultaneously.

Decisionmakers also require confidence in model-generated results to reasonably reflect the clinical environment of diseases to consider the results when making medical decisions or health care resource allocation. However, uncertainty around key model inputs and structure can put that confidence at risk. To increase confidence in the model results, best practices recommend transparency in the model’s construction, validation of the model’s ability to reproduce observed real-world outcomes, and reporting and evaluation of parameter uncertainty through sensitivity analyses [13–15]. Our objective was to create a comprehensive decision-analytic model that simulates the dynamics of multiple infections in PWIDs. In this paper, we sought to describe in detail the structure and validation of a **M**ultimorbidit**Y** model to **R**educe **I**nfections **A**ssociated with **D**rug use (**MYRIAD**) that reflect national estimates in the US to be used for future policy analyses.

## Methods

### Model description

#### Overview

**Fig. 1:**
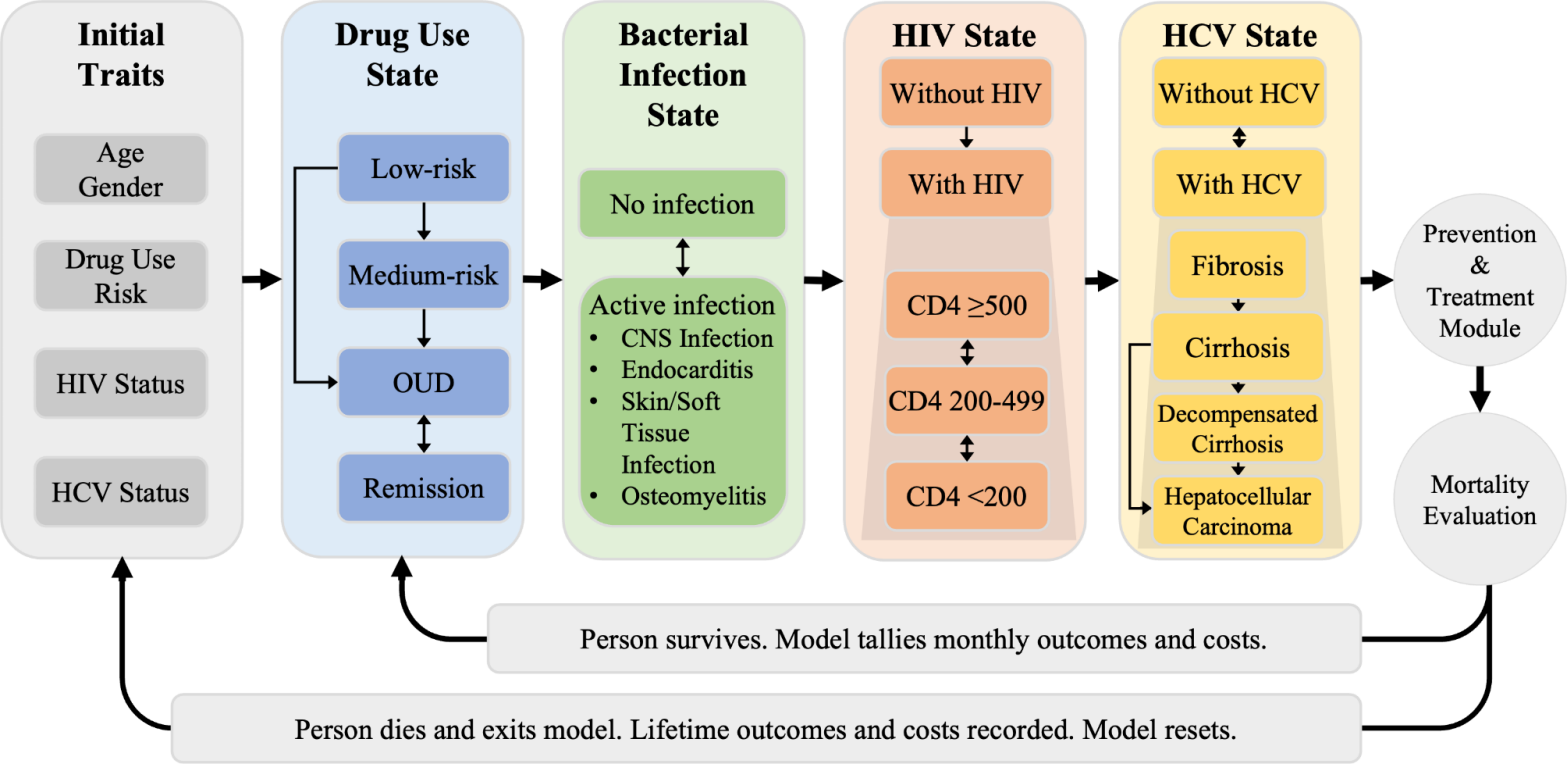
Schema for the Multimorbidity Model to Reduce Infections Associated with Drug Use (MYRIAD). Notes: HCV: hepatitis C virus; OUD: opioid use disorder; CNS: central nervous system. All persons in the model start in an active injection drug use state within the “low-risk,” “medium-risk,” and “OUD” states

MYRIAD is a microsimulation model of drug use and serious infections related to specific injection behaviors (e.g., needle sharing or reuse, especially without effective HIV pre-exposure prophylaxis, or introduction of skin or oral flora via injection) that is programmed in the R computer language (version 4.2.1) [16]. The model includes 4 health conditions: drug use behavior, serious bacterial infections, HIV, and HCV (Fig. 1). Each health condition contains many possible health states that a person can be in for that condition each month. The model creates a simulated person and assigns it an age, gender, and different health states within each of the 4 health conditions based in probabilities. No one enters the model with an active bacterial infection. The model will then increase the person’s age each month and may change the health state within each health condition for that month. This change is based on probabilities from data derived from the published literature (Table 1). Additional details on input derivations are in Supplement A. All simulated persons have a probability of death each month that is based on their age, gender, and health state they are in within each of the 4 health conditions. The model will increase the person’s age and assess changes in their health states until that person is determined to die in a particular month or until that person reaches the end of pre-set duration. The model tracks individual clinical outcomes from model start until death or when the pre-set duration is reached. We repeat the process to create a hypothetical cohort in line with recent estimates of active IDU prevalence in the US; active IDU was defined as having injected drugs within the past year [17].

**Table 1:** Model inputs to estimate health outcomes of infections associated with active injection drug use in the United States^a^

Parameter	Estimate		Source
Population			
Initial calendar year	2015		^b^
Time horizon, months	60		^b^
Proportion female (%)	32.5		[24]
Initial age, mean years (SD)	30 (2.5)		^b^
Drug Use Behavior			
Initial Opioid Use Proportion (%)			[24]
Low-risk state	62.9		
Medium-risk state	2.1		
OUD	35.0		
Monthly Transition Probabilities (%)			
Low to Medium	-^c^		
Low to OUD	-^c^		
Medium to OUD	-^c^		
OUD to Remission	2.19		^d^
Remission to OUD	5.75		[38]
Serious Bacterial Infections	**IDU**	**Non-IDU**	
Monthly Transition Probabilities			
Bacterial infection incidence (%), varies by calendar year	0.6915–0.8672	0.0262–0.0284	[4]
Infection proportion (%)			[4]
CNS infection	2.3	0.9	
Endocarditis	5.4	1.0	
Osteomyelitis	6.3	3.6	
Skin/soft tissue infection	86.0	94.6	
Cure from bacterial infection (%)	80.9	97.4	[25]
HIV	**IDU**	**Non-IDU**	
Initial HIV Probabilities			
HIV prevalence (%)	3.0	0.4	[26]
CD4 distribution (%)			[10, 27]
High CD4 (≥ 500 cells/mm^3^)	30.5	41.0	
Medium CD4 (200–499 cells/mm^3^)	29.9	16.0	
Low CD4 (< 200 cells/mm^3^)	38.6	43.0	
Aware of status if HIV positive (%)	92.7	83.5	[26]
On ART (%)	47.7	57.5	[28]
Monthly Transition Probabilities			
Monthly HIV incidence (%), varies by calendar year	0.0355–0.0382	0.0010–0.0012	[26]
Aware of status if HIV positive (%)	19.6	14.4	[26]
Start ART (%)	5.3	6.9	[28]
Stop ART (%)	1.7	1.0	[28]
CD4 Transitions (%)	**On ART**	**Off ART**	[10]
High to Medium CD4	1.14	1.24	
High to Low CD4	0.01	-	
Medium to High CD4	1.61	-	
Medium to Low CD4	0.64	7.00	
Low to Medium CD4	0.03	-	
Low to High CD4	2.66	-	
HCV	**IDU**	**Non-IDU**	
Initial HCV Probabilities			
HCV prevalence (%)	33.5	1.1	[5, 20]
Liver fibrosis stage for people starting with HCV (%)			[20]
F0	27.2		
F1	33.4		
F2	17.1		
F3	11.1		
F4	9.6		
Decompensated cirrhosis	1.4		
Hepatocellular carcinoma	0.2		
Monthly Transition Probabilities			
Monthly HCV incidence (%), varies by calendar year	0.1359–0.2024	0.0002–0.0005	[29]
% of acute HCV that clear spontaneously	2.4		[35]
Liver fibrosis transitions (%)	**HCV+**	**HCV-**	[20]
F0 to F1	0.9386	-	
F1 to F2	0.7105	-	
F2 to F3	1.0316	-	
F3 to F4	1.0222	-	
F4 to decompensated cirrhosis	0.2449	0.0669	
F4 to hepatocellular carcinoma	0.1174	0.0418	
Decompensated cirrhosis to hepatocellular carcinoma	0.5851		
Monthly Mortality Probabilities^e^			
Background Mortality	Varies by age and sex		[18]
Drug Use Mortality (%), varies by calendar year			[24]
Low-risk state	0.0052–0.0070		
Medium-risk state	0.1057–0.2580		
OUD	0.0166–0.0355		
Bacterial Infection Mortality (%)			[4]
CNS infection	3.6		
Endocarditis	5.8		
Osteomyelitis	1.5		
Skin/soft tissue infection	1.5		
HIV Mortality (%)	**On ART**	**Off ART**	[19, 21–23]
High CD4 state	0.0013	0.0033	
Medium CD4 state	0.0024	0.0155	
Low CD4 state	0.0102	0.1936	
HCV Mortality (%)			[20]
Decompensated cirrhosis	1.0		
Hepatocellular carcinoma	4.5		

^a^Active injection drug use was defined as having injected drugs within the past year

^b^User-defined

^c^Data derived from calibration

^d^Unpublished data from Opioid Policy simulation model

^e^Overall monthly mortality risks were calculated by converting all relevant mortality probabilities to rates, summing the rates, and then converting the summative rate back to a probability

SD = standard deviation; OUD = opioid use disorder; IDU = injection drug use; CNS = central nervous system

#### Mortality

After the model determines people’s final health state within each health condition for the month, it estimates the probability of death for that month. People are assigned a background mortality based on age and sex, derived using cause-specific mortality from the National Center for Health Statistics and removing mortality from substance use, IDU-related bacterial infections, HIV, and HCV since they were estimated elsewhere in the model [18]. Additional mortality rates are added onto the background mortality for each person based on their drug use behavior, type of bacterial infection, CD4 state, and liver fibrosis state (Table 1) [19–23].

#### Drug use behavior

People are assigned one of four health states regarding drug use (focusing on opioid use): “Low-risk” (i.e., prescription opioid misuse without an OUD diagnosis), “Medium-risk” (i.e., illicit opioid use such as heroin without OUD), “OUD” (OUD with prescription or illicit opioids), and “Remission.” People can transition between different states each month as described in Fig. 1. Once people reach the “OUD” state, they can only transition to the “Remission” state and vice versa (indicating relapse). No one starts out in the “Remission” state. Each opioid use state confers additional monthly mortality risk due to drug overdose. Data on the monthly transition between states and additional mortality (in excess to background mortality) within each state were derived from model calibration and the published literature (Table 1) [24]. Apart from drug use and drug-related disorders (i.e., OUD), which informs the risk of fatal drug overdose in the model, we also account for the route of drug administration. People are also assigned an IDU state of “None,” “Active,” and “Former.” An “Active” state confers additional risk of acquiring infections from the act of injection. In addition, those with IDU have a lower likelihood of starting antiretroviral therapy (ART) in people with HIV compared to those not in the “Active” state. People in the “None” and “Former” states – which can include people who orally ingest, snort, or smoke drugs – have the same probability of acquiring infections regardless of their drug use state. As this model focuses on PWID, we assume all people start in the “Active” IDU state. People who move into the “Remission” opioid use state also move into the “Former” IDU state.

#### Serious bacterial infections

People can be in either an “Active” serious bacterial infection state or have “None.” They enter the model in the “None” state. For people with an “Active” serious bacterial infection, they are assigned one of 4 types of infections: “CNS Infection,” “Endocarditis,” “Osteomyelitis,” and “Skin and Soft Tissue Infection.” The type of infection confers an additional mortality risk only for the month in which the infection occurs. The probability of acquiring or being cured of an infection as well as the type of infection depends on the current IDU state; data were derived from published literature (Table 1) [4, 25]. Only one bacterial infection can be experienced in a given month, although simulated people remain at risk for additional infections in subsequent months.

#### HIV

People enter the model in a “With HIV” or “Without HIV” state, informed from published national HIV prevalence data (Table 1) [26]. Those in the “Without HIV” state have a monthly probability of moving to the “With HIV” state based on their IDU state with inputs derived from national HIV incidence estimates [26]. People in the “With HIV” state cannot return to the “Without HIV” state. Those in the “With HIV” state are assigned a CD4 state of “High” (CD4 ≥ 500 cells/mm^3^), “Medium” (CD4 200–499 cells/mm^3^), or “Low” (CD4 < 200 cells/mm^3^); the current CD4 state determines an additional monthly mortality risk due to HIV. People can transition between CD4 states based on if they are receiving ART – those not on ART can only transition to a lower CD4 state and those on ART can transition to lower or higher CD4 states based on published data [10, 27]. The likelihood of starting or stopping ART depends on a person’s IDU state as well as if they are aware of their HIV status since only those aware of their HIV state can start ART [26, 28].

#### HCV

People enter the model in either a “With HCV” or “Without HCV” state, and they transition monthly between the states based on their IDU state (Table 1) [20]. Those in the “Without HCV” state have a monthly probability of moving to the “With HCV” state based on their IDU state, with inputs derived from national HCV incidence estimate [29]. When in the “With HCV” state, there is a monthly probability for transitioning in the Liver state, which uses the METAVIR score to stage liver fibrosis severity from “F0” through “F4”. Those with a Liver state of “F4” incur a probability of developing decompensated cirrhosis or hepatocellular carcinoma, which both confer increased liver-related mortality.

### Model validation and calibration

We used a multistep approach for model validation based on guidelines from the International Society for Pharmacoeconomics and Outcomes Research and the Society for Medical Decision Making [13].

#### Face validation

We first ensured face validity through detailed review of the model structure, underlying assumptions, and derived inputs with experts in simulation modeling of infectious diseases and opioid use who were not involved in the model’s construction.

#### Internal validation and calibration

We next internally validated the model by comparing model results to data used as model inputs for each health condition to check the validity of the model structure, and calibrated model parameters as needed to meet target estimates. For example, we isolated the HIV health condition to ensure HIV incidence and prevalence occurred as expected according to input specifications, which did not require calibration (Supplement B). However, we used national estimates of annual fatal opioid overdose rates from 2015 to 2019 to calibrate monthly transition probabilities between health states within the drug use behavior health condition.

We calibrated unknown variables within certain health conditions using the approach from Vanni et al. (Table 2) [30]. First, we identified parameters within each health condition to vary for the calibration process. Second, we selected calibration targets within each health condition. Third, we selected Pearson’s Chi-square as the goodness of fit (GOF) measure. This measure was selected to account for the level of certainty in the observed data while also balancing the complexity of the model and processing time to run the model. Fourth, we used Latin hypercube sampling as a parameter search strategy. This approach uses a probability density function and divided intervals with the same probability. A parameter value is selected within each interval for each parameter. Fifth, we established convergence criteria for each parameter being calibrated, which defines when the GOF parameters have been successfully met. We considered the convergence criteria being met when model outputs were within the 99% confidence interval of the observed calibration target values. Sixth, we established conducting 100 samples as a stopping rule for terminating the calibration process to balance identifying the ideal parameter set while also accounting for model processing time. Finally, we used the parameter sets meeting the convergence criteria and minimized the GOF measure for the external validation process.

**Table 2:** **Calibration approach** [30]

Calibration Step	Approach for MYRIAD
1. Identify parameters to vary in calibration process.	• Monthly transition probabilities between low-risk, medium-risk, and OUD states.
2. Select calibration targets.	• Fatal opioid overdose rates, 2015–2019
3. Select a goodness of fit (GOF) measure.	• Pearson’s Chi-squared
4. Select a search strategy	• Latin hypercube sampling
5. Identify acceptable GOF parameter sets (convergence criteria).	• Model outputs are within 99% confidence interval of the observed calibration target values
6. Identify the termination of the calibration process (stopping rule).	• 100 number of sample runs
7. Identify how the model calibration results and economic parameters are integrated.	• Use parameter set that minimizes GOF measure

#### Target estimates for internal validation and calibration

We calibrated the model to publicly available data. We identified parameter subsets that best fit the calibration data targets through an indirect, iterative process with Latin hypercube sampling and a GOF goal to minimize Pearson’s ${\chi }^{2}$ while within the 99% confidence interval of the calibration target data.

Target estimates for the annual fatal overdose rates from 2015 to 2019 were calculated using the number of fatal opioid overdoses identified in the Centers for Disease Control and Prevention (CDC) Wide-ranging Online Data for Epidemiologic Research (WONDER) database divided by an adjusted estimate for the number of people who misuse opioids in the US from the National Survey on Drug Use and Health (NSDUH) (Table 3) [1, 24]. Compared to published opioid overdose death rates for the general US population, which were estimated to increase from 10.4 in 2015 to 21.4 in 2020 per 100,000 people, we calibrated towards higher opioid overdose death rate targets for a population of people who misuse opioids that included people who misuse prescription opioids and/or used heroin [1]. While the number of people who misuse opioids can be challenging to estimate, this approach more accurately reflects the at-risk population for fatal opioid overdoses. We applied a multiplier from a capture-recapture analysis that more accurately reflects the prevalence of OUD to account for the underreporting of opioid misuse in the NSDUH [31]. We also accounted for the exclusion of people experiencing homelessness or incarceration in the NSDUH by estimating the number of people with substance use disorder within these populations (see Supplement Table B1) [32, 33].

**Table 3:** Calibration and validation targets for fatal opioid overdose rates in the US from 2015–2020.*

Year	Number of opioid deaths [18]	Estimated number of people who misuse opioids [24]	Fatal opioid overdose rate (99% CI)
2015	33,091	22,811,881	0.1451% (0.1383-0.1525%)
2016	42,249	21,582,291	0.1958% (0.1864-0.2061%)
2017	47,600	20,325,520	0.2342% (0.2220-0.2478%)
2018	46,802	19,081,554	0.2453% (0.2321-0.2600%)
2019	49,860	18,435,900	0.2705% (0.2531-0.2904%)
2020	68,630	17,731,936	0.3870% (0.3486-0.4351%)

* Calibration targets included years 2015–2019. Validation target included year 2020

CI = confidence interval

Target estimates for HIV incidence from 2015 to 2019 were calculated by the annual number of new HIV infections attributed to IDU divided by an estimated number of active PWID without HIV (Supplement Table B2) [5, 26]. Target estimates for HCV incidence from 2015 to 2019 were calculated by the estimated number of new HCV infections in people ages 18–40 (as a proxy for IDU) divided by the estimated age-related population of PWID (Supplement Table B3) [17, 29, 34, 35]. Target estimates for serious bacterial infection hospitalization rates from 2015 to 2017 were calculated by the estimated annual number of hospitalizations divided by the number of active PWID (Supplement Table B4) [4]. Active PWID was defined as having reported IDU within the past 12 months.

#### External validation

**Fig. 2:**
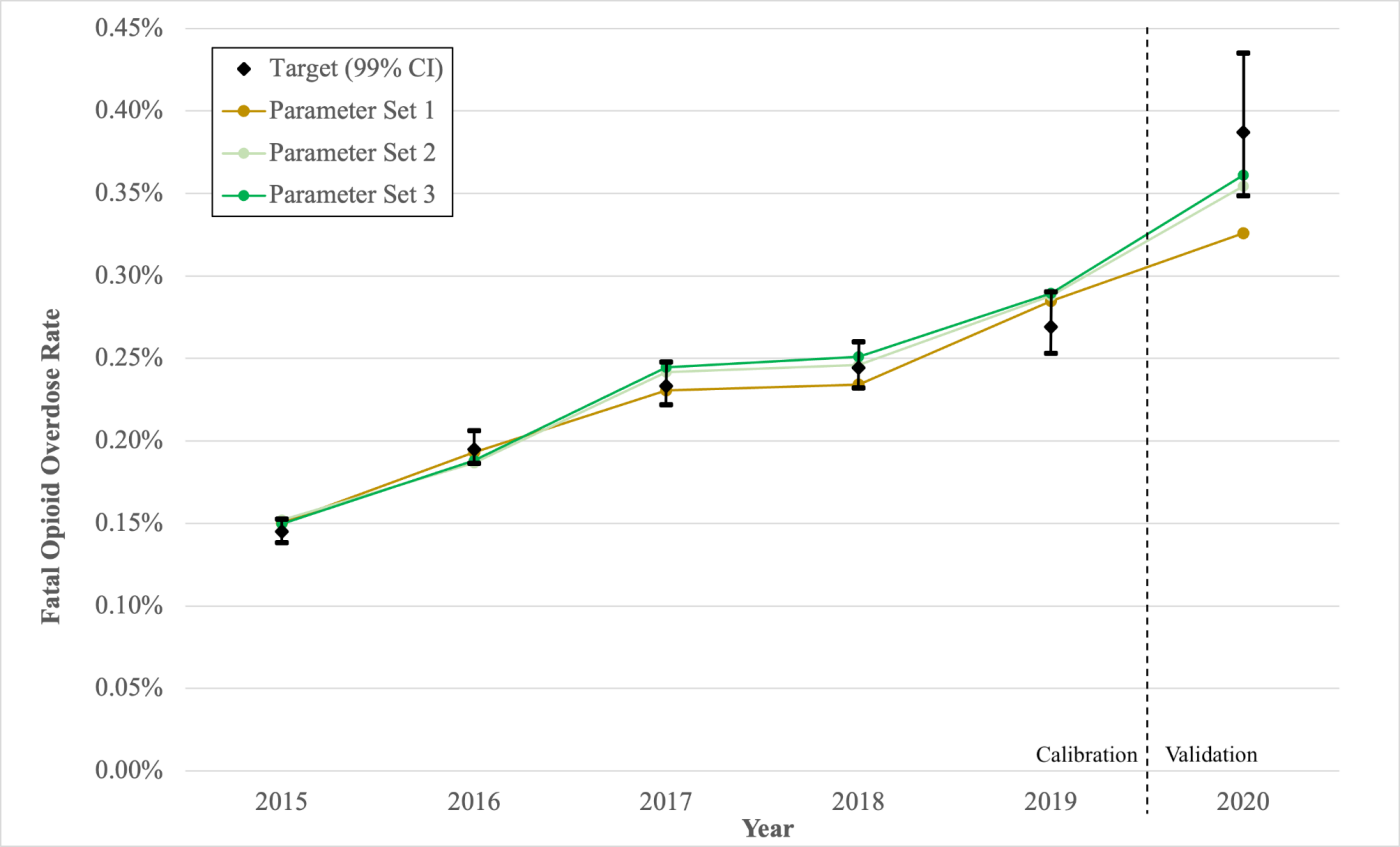
Calibration and validation of MYRIAD results to fatal opioid deaths rates among people who misuse opioids in the US from 2015–2020. Notes: CI: confidence interval

To externally validate the model, we integrated all health conditions within the model and compared model output for the year 2020 to estimated targets from the published literature for that year (Fig. 2).

### Estimates of clinical outcomes

We provided estimates of clinical outcomes over 5 years (2015–2019). To estimate the total number of fatal injection-related overdoses, we first averaged model results for the annual fatal opioid overdose rates that used parameter subsets meeting the convergence criteria. Next, we multiplied the rate by the annual estimate for the number of people who misuse opioids to calculate the number of fatal opioids overdoses each year. Lastly, we applied multipliers for the proportion of people who reported IDU upon admission for opioid use within the Treatment Episode Data Set-Admissions (TEDS-A) dataset (as a proxy for the proportion of opioid overdose deaths due to IDU) and for the proportion of injection-related deaths from opioids to calculate the total number of injection-related overdose deaths (Supplemental Table B6) [36].

The number of new HIV and HCV infections among PWID from 2015 to 2019 were estimated by applying model results for annual HIV and HCV incidence to the estimated PWID population for each year.

## Results

### Internal validation and calibration

For the drug use behavior health condition, we identified 3 independent parameters to undergo calibration. Out of 100 parameter sets derived by Latin Hypercube sampling for monthly transition probabilities among drug use behavior states, model results for 3 parameter sets fell within the 99% confidence interval of the annual opioid overdose death target data (Fig. 2). For parameter sets meeting the convergence criteria, the monthly probability of transitioning within the drug use behavior state ranged from 0.22 to 0.27% for the “Low-risk” to “Medium-risk” state, 0.06–0.32% for the “Low-risk” to “OUD” state, and 2.80–3.80% for the “Medium-risk” to “OUD” state. We identified the best-fitting parameter set with the lowest Pearson’s Chi-square (Supplement Table B5). Additional parameter sets for internal validation of severe bacterial infection hospitalization rates, HIV and HCV incidence can be found in Supplement B (Figures B1-B4). Model results for HIV and HCV incidence were within the 99% confidence interval of target estimates.

### External validation

Of the 3 parameter sets meeting the convergence criteria for fatal opioid overdose rate, 2 sets produced a model estimate that fell within the 99% confidence interval for the 2020 target rate (Fig. 2**)**.

### Estimates of clinical outcomes

From 2015 to 2019, the model estimated 223,288 fatal opioid overdoses. Using data from TEDS-A that approximated 48.9% of these deaths were injection-related and 91.2% of injection-related deaths are from opioids (Supplemental Table B7), we estimated 119,816 injection-related overdose deaths over this period. The model estimated 17,137 new HIV infections and 143,765 new HCV infections over this period as well.

## Discussion

We described the structure of a novel multimorbidity microsimulation model of injection drug use and associated infections, detailing the process for input derivation, model calibration and validation against national US data. The model produced well-fitting results to key calibration and validation targets that include fatal opioid overdose rates, HIV incidence, and HCV incidence.

The model estimated approximately 120,000 injection-related overdose deaths from 2015 to 2019. Hall et al. estimated 28,257 injection-related overdose deaths for 2018, [36] slightly above our model’s estimate of 24,957 injection-related overdose deaths for that year. Our model’s estimate of more than 17,000 new HIV infections among PWID from 2015 to 2019 is similar to HIV surveillance estimates of 18,700 (range 15,100–22,400) over the same time period (using transmission categories of IDU with or without men who have sex with men) [26]. Similarly, our model’s estimate of nearly 144,000 new HCV infections among PWID was comparable to surveillance estimates of 158,000 (range 125,000–539,000) among people aged 18–40, which serves as a proxy for new HCV infections from IDU [29].

The model’s value is in its ability to address the syndemic of drug use and associated infections by incorporating drug overdose with injection-related viral and bacterial infections, which is particularly important for PWID who may experience many infections concurrently. In the absence of preventive measures, undiagnosed asymptomatic HIV and HCV each propagate transmission of the other, and treatment delays worsen the long-term response as well as survival. This is reflected in our input parameters that incorporate higher monthly probabilities for developing a new bacterial infection, HIV, HCV infection in PWID (Table 1). As PWID seek medical care for acute symptomatic bacterial infections, an opportunity arises for earlier HIV and HCV diagnoses, linkage to care, and treatment of OUD, a key contributor to IDU. Other models of IDU have focused on a viral infection (HIV and HCV) or a bacterial infection (e.g., endocarditis), while this model integrates multiple infections with an emphasis on the multimorbidity of IDU [9–12].

The model also distinguishes substance use and substance use disorders (e.g., OUD) from the act of injection, which will allow us to look at interventions with different goals. For example, syringe service programs focus on reducing infections associated with the act of injection without addressing the drug use (i.e., harm reduction). Whereas medications for OUD would address OUD (but not necessarily non-opioid substance use disorder), where IDU may or may not decrease. This model allows us to look at the impact of medications for OUD on its own, syringe service programs on its own, and an integrated syringe service program with MOUD that could synergistically impact IDU and OUD together.

We hope that this multimorbidity model will allow us to inform health policy guidance in the US, for example, by assessing packages of interventions that can reduce drug overdose deaths (e.g., medications for OUD, safe consumptions sites), reduce harms associated with specific injection practices (e.g., syringe service programs), prevent HIV (i.e., pre-exposure prophylaxis), and improve linkage to care for chronic viral infections such as HIV and HCV. This model can also assess the impact of an intervention on multiple infections concurrently, such as the reduction of bacterial infections, HIV, and HCV with syringe service programs.

There lacks a standard approach to model validation in the development process. Best practice guidelines developed by the International Society for Pharmacoeconomics and Outcomes Research and the Society for Medical Decision Making highlight the importance of transparency and validation in model development so that decisionmakers can trust the model output when making informed healthcare decisions [13]. Other health models have transparently reported model development with a systematic approach to calibration and validation. Zang et al. reported on the development and calibration of a dynamic compartmental model of HIV transmission among 6 US cities [15].

While we used a systematic approach for selecting calibration targets outlined by Vanni et al., there is no standardized approach to model calibration and validation [30]. A standardized approach may be challenging given the limited availability of data to both calibrate and validate the model. For example, we used national estimates of annual fatal opioid overdose rates from 2015 to 2019 to calibrate the model and separately validated the model with data from 2020.

Our analysis has limitations. First, calibration targets of national estimates are derived from cross-sectional surveys that may undercount people who use drugs and PWID due to reporting and selection biases. We addressed these biases by adjusting for the number of people who misuses opioids in the US and using a 99% confidence interval for calibration targets to fall within given uncertainty of the data. Second, the model was calibrated to overall opioid overdose deaths in the US given a lack of injection-specific data. Newly published data estimating the proportion of injection-related overdoses for opioid and non-opioid use will be utilized in future analyses [36]. Third, the model does not account for differences in clinical outcomes by race/ethnicity due to differing rates of access to prevention and treatment measures, which will be the focus of a future analysis. Fourth, the model does not account for the impact of COVID-19 on IDU. In our next planned set of policy-focused analyses, we will consider this impact as well as the impact from shifts in opioid usage (e.g., increasing fentanyl use) when projecting future clinical outcomes. We can account for the accelerated overdose deaths from fentanyl using data from the CDC WONDER database, where D’Orsogna et al. found that fentanyl-related fatal overdose rates for 2020 exceeded projections by 30% [37]. Fifth, our model may undercount clinical outcomes when all people start in an active IDU state, thus not accounting for those in remission from OUD or have a history of IDU who may relapse. Our focus for this analysis were people with active IDU, but future analyses can incorporate those who relapse to better account for additional clinical and economic outcomes. Sixth, this analysis focuses on selected infections of clinical and public health interests in the US; we exclude other injection-related infections that may play a more significant role in other countries such as tuberculosis or hepatitis B. Seventh, HCV treatment for PWID is not included in the model and will be incorporated into future analyses. Eighth, as with all simulation models, the model necessarily simplifies complex biosocial processes while also retaining key parameters that drive outcomes for IDU and associated infections. We internally validated our model with experts in simulation modeling of HIV, HCV, and opioid use to maintain equipoise between the simplifying assumptions and reflecting the complex interactions between drug use and its associated infections. Finally, our calibration approach of Latin hypercube sampling risks convergence on local optima (i.e., the most favorable solution among neighboring set of solutions, but not the most favorable solution among all possible solutions). To address this, we average model results from all 3 parameter sets that met the convergence criteria.

## Conclusion

Injection drug use has converged multiple epidemics into a syndemic in the US, including opioid use, HIV, HCV, and serious bacterial infections. There are limited national data on clinical outcomes for PWID, and in the absence of clinical trials and nationally representative longitudinal observational studies of PWID, simulation models are critical in providing expected outcomes for decisionmakers. This validated and calibrated model will next be used to project clinical outcomes and costs. We hope that this microsimulation model of injection drug use will be an important tool for decision-makers; it will allow them to project long-term clinical outcomes beyond the horizon of traditional clinical studies, as well as short- and long-term costs, and thus to evaluate the cost-effectiveness of interventions that reduce drug overdoses and other harms associated with injection drug use.

## Electronic supplementary material

Below is the link to the electronic supplementary material.

Supplementary Material 1

## Data Availability

The datasets used and/or analysed during the current study are available from the corresponding author on reasonable request.
