# Supplementary material for: A multimorbidity model for estimating health outcomes from the syndemic of injection drug use and associated infections in the United States
Source: BMC Health Serv Res. 2023 Jul 17;23:760. doi: 10.1186/s12913-023-09773-1 (PMC10353126; doi:10.1186/s12913-023-09773-1)
Supplement: Supplementary file 1 — Supplementary Material 1 [file 12913_2023_9773_MOESM1_ESM.docx]

**A multimorbidity model for estimating health outcomes from injection drug use and associated infections in the United States**

John J. Chiosi^1,2^, Peter P. Mueller^3^, Jagpreet Chhatwal^2,3^, Andrea L. Ciaranello^1,2^

^1^Medical Practice Evaluation Center and Division of Infectious Diseases, Massachusetts General Hospital, Boston, Massachusetts, USA

^2^Harvard Medical School, Boston, Massachusetts, USA

^3^Institute for Technology Assessment, Massachusetts General Hospital, Boston, Massachusetts, USA

**Supplementary Data**

**Supplement A: Input Derivations**

1. **Cohort Characteristics**
   1. ***Proportion female***

Data were extracted from the National Survey of Drug Use and Health (NSDUH), 2015-2019, using variables for any injection drug (ANYNDLREC) and sex (IRSEX).^1^ Active injection drug use (IDU) was defined as any injection drug use within the past 12 months. The weighted values for national estimates were used to obtain estimated for each year and an average across the years was obtained.

**Table A1. National weighted estimates for the number of people who reported inject drug use within the past 12 months by gender from the NSDUH, 2015-2019**

| **NSDUH Year** | **Total Weighted Population with IDU in the past 12 months** | **Male** | **Female** |
| --- | --- | --- | --- |
| 2015 | 790,861 | 595,462  (75.3%) | 195,400  (24.7%) |
| 2016 | 846,005 | 557,803  (65.9%) | 288,202  (34.1%) |
| 2017 | 948,076 | 667,342  (70.4%) | 277,900  (29.3%) |
| 2018 | 828,196 | 533,815  (64.5%) | 294,382  (35.5%) |
| 2019 | 697,435 | 425,687  (61.0%) | 271,748  (39.0%) |
| **5-year Average Percentage:** | | **67.4%** | **32.5%** |

1. **Drug Use Behavior**
   1. ***Initial Opioid Use Prevalence***

Data were extracted from the 2015 NSDUH, using variables for heroin use and pain reliever misuse (HERPNRYR), and heroin and pain reliever use disorder (UDPYHRPNR).^1^ The number of people in the opioid use disorder (OUD) state was calculated from any substance use disorder of heroin and/or pain relievers as defined by UDPYHRPNR. The number of people in the low-risk opioid use state was calculated as the number of people with any pain reliever misuse subtracted by the number of people with pain reliever use disorder (without concurrent heroin use disorder). The number of people in the medium-risk state was calculated as any heroin use (regardless of pain reliever use) subtracted by heroin use disorder (regardless of pain reliever use). These values were adjusted to account for reporting and selection biases in the NSDUH.^2–4^

**Table A2. National weighted estimates for the number of people who misuse opioids by opioid use state from the 2015 NSDUH**

| **NSDUH Year** | **Low-risk** | **Medium-risk** | **OUD** |
| --- | --- | --- | --- |
| 2015 | 14,353,565 (62.9%) | 477,290  (2.1%) | 7,981,027 (35.0%) |

- 1. ***Monthly transition probabilities of opioid use state***

Parameters were estimated through calibration using Latin Hypercube Sampling as described in the main paper.

1. **Serious Bacterial Infections**
   1. ***Monthly incidence of bacterial infection***

The model uses the monthly probability of acquiring any bacterial infection according the IDU state prior to assigning the type of bacterial infection a person has. Data on the incidence and distribution of bacterial infection by IDU state were derived from McCarthy et al., which used inpatient data from the Premier Healthcare Database, a claims database of private and academic hospitals representing approximately 20% of US inpatient discharges that is weighted to reflect national data, to estimate the annual number of bacterial infections associated with substance use disorder in the US from 2012-2017.^5^ The study focused on 4 infections associated with IDU: endocarditis, osteomyelitis, central nervous system abscess, and skin/soft tissue infections. Infections and substance use disorder were identified by *International Classification of Diseases, Ninth Revision* and *Tenth Revision* (ICD-9/10) codes. Due to the lack of an ICD-9/10 code specific for IDU, the code for substance use disorder (SUD) was used as a proxy. We stratified the number of hospitalizations by infection type according to SUD status by multiplying the provided proportion of hospitalizations with a SUD diagnosis for each infection.

**Table A3. Total estimated hospitalizations in the US for selected infections by SUD status, 2012-2017**

|  | **2012** | **2013** | **2014** | **2015** | **2016** | **2017** |
| --- | --- | --- | --- | --- | --- | --- |
| *Endocarditis* | *12,415* | *12,081* | *11,600* | *11,686* | *11,760* | *12,939* |
| Non-SUD | 9,944 | 9,206 | 8,538 | 8,215 | 7,926 | 7,841 |
| SUD | 2,471 | 2,875 | 3,062 | 3,471 | 3,834 | 5,098 |
| *Osteomyelitis* | *39,246* | *39,623* | *40,537* | *40,014* | *47,458* | *35,773* |
| Non-SUD | 36,028 | 36,057 | 36,037 | 35,532 | 42,475 | 29,835 |
| SUD | 3,218 | 3,566 | 4,500 | 4,482 | 4,983 | 5,938 |
| *CNS abscess* | *7,801* | *7,722* | *7,480* | *7,831* | *8,764* | *9,144* |
| Non-SUD | 6,600 | 6,494 | 6,171 | 6,351 | 7,046 | 6,968 |
| SUD | 1,201 | 1,228 | 1,309 | 1,480 | 1,718 | 2,176 |
| *Skin/Soft Tissue infection* | *845,300* | *822,250* | *804,036* | *797,066* | *862,065* | *860,161* |
| Non-SUD | 787,820 | 758,115 | 735,693 | 725,330 | 787,065 | 779,306 |
| SUD | 57,480 | 64,136 | 68,343 | 71,736 | 75,000 | 80,855 |
| *Any SBI* | *904,762* | *881,676* | *863,653* | *856,597* | *930,047* | *918,017* |
| Non-SUD | 840,391 | 809,871 | 786,439 | 775,429 | 844,513 | 823,949 |
| SUD | 64,371 | 71,805 | 77,214 | 81,168 | 85,534 | 94,068 |

To calculate the rate of hospitalizations among people with do and do not inject drugs, we estimated the prevalence of PWID within the US. We applied the estimated prevalence of IDU within the past 12 months in the US from Bradley et al. (0.41%, 95% CI 0.35-0.47%) to the total US population age ≥18 years

**Table A4. Total estimated number of PWID in the US, 2012-2017**

|  | **2012** | **2013** | **2014** | **2015** | **2016** | **2017** |
| --- | --- | --- | --- | --- | --- | --- |
| ***Total population ≥18 years*** | 240,144,241 | 242,470,820 | 245,201,076 | 247,813,911 | 249,454,442 | 255,797,692 |
| *Basecase estimate = 0.41%* | | | | | | |
| IDU | 984,591 | 994,130 | 1,005,324 | 1,016,037 | 1,022,763 | 1,048,771 |
| Non-IDU | 239,159,649 | 241,476,689 | 244,195,752 | 246,797,874 | 248,431,678 | 254,748,921 |
| *Lower bound = 0.35%* | | | | | | |
| IDU | 1,128,678 | 1,139,613 | 1,152,445 | 1,164,725 | 1,172,436 | 1,202,249 |
| Non-IDU | 239,015,563 | 241,331,207 | 244,048,631 | 246,649,186 | 248,282,006 | 254,595,443 |
| *Upper bound = 0.47%* | | | | | | |
| IDU | 840,505 | 848,648 | 858,204 | 867,349 | 873,091 | 895,292 |
| Non-IDU | 239,303,736 | 241,622,172 | 244,342,872 | 246,946,562 | 248,581,351 | 254,902,400 |

The hospitalization rate for a bacterial infection by IDU status was then calculated with the number of IDU-related and non-IDU-related hospitalizations for an infection divided by the total IDU and non-IDU populations in the US. The annual hospitalization rates were then converted to monthly probabilities.

Monthly probabilities for calendar years 2015-2017 were directly incorporated into the model, while monthly probabilities for calendar years 2018-2019 were imputed from an assumed linear trend line using data from years 2012-2017.

**Table A5. Hospitalization rates for bacterial infection by IDU status, 2012-2017.**

|  | **2012** | **2013** | **2014** | **2015** | **2016** | **2017** | **2018** | **2019** |
| --- | --- | --- | --- | --- | --- | --- | --- | --- |
| *Annual rate (per 100,000 people)* | | | | | | | | |
| IDU | 6,537.8 | 7,222.9 | 7,680.5 | 7,988.7 | 8,363.1 | 8,969.3 | - | - |
| Non-IDU | 351.4 | 335.4 | 322.1 | 314.2 | 339.9 | 323.4 | - | - |
| *Monthly probability* | | | | | | | | |
| **IDU** | 0.5619% | 0.6228% | 0.6637% | 0.6914% | 0.7252% | 0.7801% | 0.8167% | 0.8575% |
| **Non-IDU** | 0.0293% | 0.0280% | 0.0269% | 0.0262% | 0.0284% | 0.0270% | 0.0287% | 0.0287% |

- 1. ***Distribution of bacterial infections***

The distribution of bacterial infections by SUD status (shown in Table A4) were calculated for each year and an average proportion for calendar years 2015-2017 was used as data inputs.

- 1. ***Probability of cure***

Data on probability of cure calculated as the complement to the proportion of patient-directed discharges (i.e., hospital discharge against medical advice) by OUD status from Kim et al.^6^

**Table A6. Probability of cure**

|  | **Proportion of AMA** | **Monthly probability of cure** |
| --- | --- | --- |
| Non-OUD | 2.6% | 97.4% |
| OUD | 19.1% | 80.9% |

1. **HIV**
   1. ***HIV prevalence***

HIV prevalence was estimated for the year 2015. A lifetime IDU prevalence of 2.6% was applied to a general US population age ≥18 years to estimate the total adult IDU and non-IDU population.^7^ The number of diagnosed or undiagnosed HIV cases were obtained from national estimates by the CDC.^8^ Cases attributed to IDU were from the IDU and IDU+MSM transmission categories, non-IDU cases were from all other transmission categories.

**Table A7. HIV prevalence by IDU status in 2015**

|  | **IDU** | **Non-IDU** |
| --- | --- | --- |
| Number of HIV cases (diagnosed or undiagnosed) | 190,100 | 908,700 |
| US population ≥18 years | 6,442,517 | 241,346,594 |
| HIV prevalence (%) | 2.95 | 0.38 |

- 1. ***HIV incidence***

HIV incidence was calculated using national incidence estimates from the CDC divided by a population of non-HIV population stratified by IDU state.^8^

**Table A8. HIV incidence by IDU status**

|  | **2015** | **2016** | **2017** | **2018** | **2019** |
| --- | --- | --- | --- | --- | --- |
| ***IDU*** |  |  |  |  |  |
| HIV incidence | 3,700 | 3,600 | 3,600 | 3,900 | 3,900 |
| HIV prevalence | 190,100 | 188,700 | 187,700 | 186,900 | 186,500 |
| Total population | 1,015,935 | 1,022,908 | 1,033,489 | 1,040,642 | 1,046,614 |
| Rate (per 100,000) | 448.0 | 431.5 | 425.6 | 456.8 | 453.4 |
| ***Non-IDU*** |  |  |  |  |  |
| HIV incidence | 34,100 | 34,300 | 33,100 | 32,300 | 30,900 |
| HIV prevalence | 908,700 | 932,900 | 956,000 | 978,400 | 999,900 |
| Total population | 246,773,176 | 248,466,864 | 251,037,006 | 252,774,555 | 254,225,124 |
| Rate (per 100,000) | 13.9 | 13.9 | 13.2 | 12.8 | 12.2 |

- 1. ***CD4 count distribution***

The distribution of CD4 among PWID were obtained from the HIV Research Network via Krebs et al.^9^ For people without IDU who acquire HIV, the CD4 distribution was obtained from Long et al.^10^

- 1. ***Probability of HIV diagnosis***

The likelihood of a person with HIV being diagnosed was derived from national estimated of the proportion of HIV cases that are diagnosed from the CDC.^8^ We used the proportion of diagnosed HIV cases in 2015 for the initial distribution and the average proportion from 2015-2019 stratified by IDU status for new HIV infections.

- 1. ***Probability of starting and stopping antiretroviral therapy***

The probability of starting and stopping antiretroviral therapy (ART) were from national estimates from the Medical Monitoring project from 2015-2018, stratified by IDU status.^11^

- 1. ***CD4 transitions***

The monthly probabilities of transitioning between CD4 groups, were obtained from the HIV Research Network via Krebs et al.^9^ This data differed based on whether a person was receiving ART.

1. **HCV**
   1. ***HCV prevalence***

HCV prevalence among PWID was calculated using the prevalence rate if HCV infection of 43,126 per 100,000 PWID from Lansky et al. and multiplying by the chronic HCV infection ratio (percentage of positive HCV antibodies with positive HCV RNA) estimated by Kabiri et al (77.6%).^7,12^

- 1. ***HCV incidence***

HCV incidence was calculated using national estimates of acute HCV cases (using HCV cases in people aged 18-40 as a proxy for PWID) from the CDC divided by a population stratified by IDU state.^13,14^

**Table A9. HCV incidence by IDU status**

|  | **Estimated Acute HCV cases** | | | **Estimated number of PWID** | | **Incidence** | |
| --- | --- | --- | --- | --- | --- | --- | --- |
|  | **Overall** | **Age 18-40*** | **All others** | **PWID** | **Non-PWID** | **PWID** | **Non-PWID** |
| 2015 | 33,900 | 24,900 | 9,000 | 1,538,263 | 314,660,243 | 1.619% | 0.003% |
| 2016 | 41,200 | 29,300 | 11,900 | 1,548,193 | 316,691,411 | 1.893% | 0.004% |
| 2017 | 44,700 | 31,000 | 13,700 | 1,564,840 | 319,439,567 | 1.981% | 0.004% |
| 2018 | 50,300 | 34,700 | 15,600 | 1,576,540 | 321,326,490 | 2.201% | 0.005% |
| 2019 | 57,500 | 38,100 | 19,400 | 1,586,103 | 323,111,692 | 2.402% | 0.006% |
| 2020 | 66,700 | 40,400 | 26,300 | 1,595,729 | 324,973,579 | 2.532% | 0.008% |

* Acute HCV cases in ages 18-40 were used as a proxy for cases in PWID in the 2020 National Hepatitis Surveillance.

- 1. ***Probability of HCV cure***

The proportion of acute HCV cases that clear spontaneously annually was estimated from Grebely et al.^15^

- 1. ***Initial liver fibrosis state***

The initial liver fibrosis state for people entering the model with HCV were obtained from Kabiri et al.^12^

- 1. ***Probability of liver fibrosis state transition***

The monthly progression of liver fibrosis state in people with HCV were obtained from Kabiri et al.^12^

1. **Mortality**
   1. **Background Mortality**

Age- and sex-stratified background mortality were derived from life tables from the National Vital Statistics Systems with cause-specific mortality removed for opioid use, HIV, selected bacterial infections, and hepatitis C.

**Table A10. Age- and sex-stratified monthly background mortality probability.**

| **Age** | **Male** | **Female** |
| --- | --- | --- |
| 0 | 0.000535030 | 0.000449818 |
| 1 | 0.000037657 | 0.000029432 |
| 2 | 0.000023510 | 0.000018997 |
| 3 | 0.000018991 | 0.000013683 |
| 4 | 0.000013900 | 0.000010778 |
| 5 | 0.000013209 | 0.000009531 |
| 6 | 0.000011589 | 0.000009950 |
| 7 | 0.000009690 | 0.000008538 |
| 8 | 0.000010692 | 0.000008058 |
| 9 | 0.000010384 | 0.000006930 |
| 10 | 0.000010899 | 0.000006985 |
| 11 | 0.000010609 | 0.000008203 |
| 12 | 0.000011648 | 0.000009339 |
| 13 | 0.000015860 | 0.000012337 |
| 14 | 0.000021590 | 0.000014447 |
| 15 | 0.000027933 | 0.000014853 |
| 16 | 0.000038747 | 0.000019977 |
| 17 | 0.000054966 | 0.000023284 |
| 18 | 0.000071846 | 0.000030154 |
| 19 | 0.000085445 | 0.000033625 |
| 20 | 0.000093163 | 0.000034228 |
| 21 | 0.000104205 | 0.000038218 |
| 22 | 0.000112044 | 0.000038749 |
| 23 | 0.000112925 | 0.000040217 |
| 24 | 0.000117593 | 0.000042420 |
| 25 | 0.000119716 | 0.000046224 |
| 26 | 0.000118110 | 0.000046762 |
| 27 | 0.000124918 | 0.000050386 |
| 28 | 0.000125280 | 0.000053916 |
| 29 | 0.000135123 | 0.000056596 |
| 30 | 0.000129512 | 0.000060768 |
| 31 | 0.000137492 | 0.000063773 |
| 32 | 0.000142170 | 0.000070695 |
| 33 | 0.000145073 | 0.000073673 |
| 34 | 0.000152568 | 0.000076969 |
| 35 | 0.000151455 | 0.000078083 |
| 36 | 0.000158803 | 0.000090166 |
| 37 | 0.000165128 | 0.000090137 |
| 38 | 0.000168719 | 0.000098474 |
| 39 | 0.000176649 | 0.000098901 |
| 40 | 0.000177881 | 0.000107704 |
| 41 | 0.000195285 | 0.000120137 |
| 42 | 0.000209416 | 0.000132495 |
| 43 | 0.000219365 | 0.000140090 |
| 44 | 0.000242979 | 0.000154648 |
| 45 | 0.000251012 | 0.000164625 |
| 46 | 0.000285355 | 0.000184440 |
| 47 | 0.000301934 | 0.000197657 |
| 48 | 0.000334407 | 0.000222925 |
| 49 | 0.000378053 | 0.000241222 |
| 50 | 0.000408625 | 0.000262990 |
| 51 | 0.000455092 | 0.000286912 |
| 52 | 0.000495924 | 0.000318245 |
| 53 | 0.000548641 | 0.000347136 |
| 54 | 0.000602780 | 0.000368855 |
| 55 | 0.000636171 | 0.000391388 |
| 56 | 0.000704509 | 0.000436561 |
| 57 | 0.000769753 | 0.000466954 |
| 58 | 0.000818233 | 0.000499924 |
| 59 | 0.000890915 | 0.000530444 |
| 60 | 0.000942500 | 0.000554643 |
| 61 | 0.001025089 | 0.000603826 |
| 62 | 0.001104985 | 0.000657288 |
| 63 | 0.001210396 | 0.000708700 |
| 64 | 0.001254932 | 0.000753593 |
| 65 | 0.001321549 | 0.000806661 |
| 66 | 0.001423078 | 0.000886492 |
| 67 | 0.001509315 | 0.000971669 |
| 68 | 0.001566675 | 0.001021589 |
| 69 | 0.001817644 | 0.001195693 |
| 70 | 0.001905008 | 0.001277171 |
| 71 | 0.002138215 | 0.001419132 |
| 72 | 0.002262435 | 0.001559130 |
| 73 | 0.002512001 | 0.001713147 |
| 74 | 0.002730191 | 0.001857252 |
| 75 | 0.002970607 | 0.002058929 |
| 76 | 0.003253759 | 0.002278223 |
| 77 | 0.003559192 | 0.002523880 |
| 78 | 0.003925244 | 0.002794599 |
| 79 | 0.004303367 | 0.003131631 |
| 80 | 0.004772468 | 0.003468489 |
| 81 | 0.005413344 | 0.003874935 |
| 82 | 0.005887471 | 0.004377174 |
| 83 | 0.006534743 | 0.004874856 |
| 84 | 0.007311953 | 0.005440980 |
| 85 | 0.007916233 | 0.006011048 |
| 86 | 0.008952901 | 0.006860804 |
| 87 | 0.010141336 | 0.007742214 |
| 88 | 0.011113141 | 0.008683902 |
| 89 | 0.012297409 | 0.009780885 |
| 90 | 0.013601341 | 0.011042032 |
| 91 | 0.015038681 | 0.012285537 |
| 92 | 0.016405485 | 0.013703569 |
| 93 | 0.018274825 | 0.015408261 |
| 94 | 0.019318236 | 0.016739831 |
| 95 | 0.021135113 | 0.018631912 |
| 96 | 0.021977613 | 0.020379202 |
| 97 | 0.023222399 | 0.021931404 |
| 98 | 0.024785516 | 0.024980146 |
| 99 | 0.026221238 | 0.025487445 |
| 100 | 1.00000000 | 1.00000000 |

- 1. **Opioid Overdose Mortality**

Opioid overdose deaths were obtained from the Multiple Causes of Death database of CDC Wide-ranging Online Data for Epidemiologic Research (WONDER). Data are based on death certificates for US residents within the 50 states and DC. They include county-level national mortality and population data spanning the years 1999-2020. These data were limited to age 18 years and older.

Death from illicit opioid use was determined if any of the following ICD-10 codes were used as a contributing cause of death: T40.0 (opium), T40.1 (heroin), T40.4 (other synthetic narcotic, including fentanyl). Death from prescription opioids was calculated as the difference between all opioid deaths and illicit opioid deaths. Since there is no category for opioid deaths due to OUD in the CDC WONDER database, it was assumed the proportion of deaths attributed to OUD was equal to the proportion of OUD out of the total prescription opioid misuse/heroin use population in the NSDUH.

Annual probability of death was calculated with the number of deaths by drug use state over the estimated population within each drug use state per year.

**Table A11. Annual probability of opioid overdose probability by opioid use state**

| **Year** | **Estimated Number of Opioid Deaths** | | | | **Annual Probability of Opioid Overdose Deaths** | | |
| --- | --- | --- | --- | --- | --- | --- | --- |
|  | **Total** | **Prescription Opioids** | **Illicit Opioids** | **OUD** | **Prescription Opioids** | **Illicit Opioids** | **OUD** |
| 2015 | 33,091 | 11,230 | 6,022 | 15,840 | 0.078% | 1.262% | 0.198% |
| 2016 | 42,249 | 11,302 | 9,359 | 21,589 | 0.084% | 1.650% | 0.284% |
| 2017 | 47,600 | 10,173 | 8,755 | 28,672 | 0.081% | 2.097% | 0.392% |
| 2018 | 46,802 | 8,145 | 12,739 | 25,919 | 0.071% | 2.341% | 0.366% |
| 2019 | 49,860 | 7,287 | 15,949 | 26,624 | 0.063% | 3.052% | 0.426% |
| 2020 | 68,630 | 7,039 | 24,794 | 36,767 | 0.068% | 3.448% | 0.546% |

- 1. **Bacterial Infection Mortality**

Mortality from bacterial infection were obtained from McCarthy et al. and stratified by infection type.^5^

**Table A12. Proportion with in-hospital death by infection**

| **Infection** | **2015** | **2016** | **2017** | **3-yr average** |
| --- | --- | --- | --- | --- |
| Endocarditis | 6.5% | 5.5% | 5.3% | 5.8% |
| Osteomyelitis | 1.5% | 1.4% | 1.6% | 1.5% |
| Central Nervous System Infection | 4.0% | 3.6% | 3.3% | 3.6% |
| Skin/Soft Tissue Infection | 1.3% | 1.5% | 1.7% | 1.5% |

- 1. **HIV Mortality**

HIV-specific mortality is stratified by CD4 state and whether a person is taking antiretroviral therapy. Inputs were derived using data from the NA-ACCORD,^16^ MACS,^17^ and CAUSAL.^18^

**Table A13. Monthly Probability of HIV-related Death by CD4 and treatment state**

|  | **Off-ART** | **On-ART** |
| --- | --- | --- |
| CD4 >500 | 0.0033% | 0.0013% |
| CD4 200-499 | 0.0155% | 0.0024% |
| CD4 <200 | 0.1936% | 0.0102% |

- 1. **HCV Mortality**

HCV-specific mortality from hepatocellular carcinoma and decompensated cirrhosis is from Kabiri et al.^12^

**Supplement B: Model Calibration, Model Validation, and Outcome Estimates**

**Table B1. Estimated number of people who misuse opioids in the US, 2015-2020.**

| **Year** | **Unadj. Estimate^a^** | **Lower 95% CI** | **Upper 95% CI** | **Adj. Estimate^b^** | **Lower Bound** | **Upper Bound** |
| --- | --- | --- | --- | --- | --- | --- |
| **2015** | 12,681,912 | 11,834,947 | 13,528,877 | 22,811,881 | 21,696,430 | 23,927,332 |
| **2016** | 11,915,797 | 11,091,001 | 12,740,593 | 21,582,291 | 20,496,036 | 22,668,546 |
| **2017** | 11,142,456 | 10,297,490 | 11,987,422 | 20,325,520 | 19,212,701 | 21,438,339 |
| **2018** | 10,328,452 | 9,508,587 | 11,148,317 | 19,081,554 | 18,001,794 | 20,161,314 |
| **2019** | 10,113,771 | 9,153,039 | 11,074,503 | 18,435,900 | 17,170,618 | 19,701,182 |
| **2020** | 9,464,162 | 7,977,671 | 10,950,653 | 17,731,936 | 15,774,230 | 19,689,642 |

CI = confidence interval

^a^Unadjusted estimates derived from the annual National Survey of Drug Use and Health (NSDUH) data.

^b^Adjusted estimates accounted for people experiencing homelessness and incarceration that are excluded from the NSDUH.

**Table B2. Target estimates for HIV incidence among active PWID in the US from 2015-2019.**

|  |  | **PWID Population^a^** | | | **Number of PWID with HIV**^b^ | | | **Number of new HIV infections from IDU^b^** | | | **Calculated HIV incidence among active PWID** | | |
| --- | --- | --- | --- | --- | --- | --- | --- | --- | --- | --- | --- | --- | --- |
| **Yr.** | **Total Pop. ≥18y** | **Estimate** | **LB** | **UB** | **Estimate** | **LB** | **UB** | **Estimate** | **LB** | **UB** | **Estimate** | **LB** | **UB** |
| 2015 | 247,789,111 | 1,015,935 | 867,262 | 1,164,609 | 190,100 | 185,900 | 194,300 | 3,700 | 3,200 | 4,300 | 0.4480% | 0.3270% | 0.5233% |
| 2016 | 249,489,772 | 1,022,908 | 873,214 | 1,172,602 | 188,700 | 184,500 | 193,000 | 3,600 | 2,900 | 4,200 | 0.4315% | 0.2935% | 0.5061% |
| 2017 | 252,070,495 | 1,033,489 | 882,247 | 1,184,731 | 187,700 | 183,300 | 192,000 | 3,600 | 2,900 | 4,300 | 0.4256% | 0.2896% | 0.5110% |
| 2018 | 253,815,197 | 1,040,642 | 888,353 | 1,192,931 | 186,900 | 172,500 | 191,300 | 3,900 | 3,100 | 4,800 | 0.4568% | 0.3038% | 0.5651% |
| 2019 | 255,271,738 | 1,046,614 | 893,451 | 1,199,777 | 186,500 | 182,000 | 191,000 | 3,900 | 3,000 | 4,800 | 0.4534% | 0.2948% | 0.5610% |

PWID = people who inject drugs; IDU = injection drug use; LB = lower bound; UB = upper bound

^a^PWID population was estimated from a prevalence of IDU within the past 12 months of 0.41% (95% CI 0.35%-0.47%)^7^

**^b^**Includes IDU and IDU+MSM categories^8^

**Table B3. Target estimates for HCV incidence among active PWID in the US from 2015-2019.**

|  |  | **PWID Population^a^** | | | **Number of acute HCV infections from IDU** | | | **Calculated HCV incidence among active PWID** | | |
| --- | --- | --- | --- | --- | --- | --- | --- | --- | --- | --- |
| **Yr.** | **Total Pop.** | **Estimate** | **LB** | **UB** | **Estimate** | **LB** | **UB** | **Estimate** | **LB** | **UB** |
| 2015 | 316,198,506 | 1,538,263 | 2,076,655 | 12,015,543 | 24,900 | 19,701 | 84,893 | 1.619% | 0.164% | 4.088% |
| 2016 | 318,239,604 | 1,548,193 | 2,090,060 | 12,093,105 | 29,300 | 23,221 | 100,061 | 1.893% | 0.192% | 4.787% |
| 2017 | 321,004,407 | 1,564,840 | 2,106,620 | 12,198,167 | 31,000 | 24,519 | 105,655 | 1.981% | 0.201% | 5.015% |
| 2018 | 322,903,030 | 1,576,540 | 2,117,857 | 12,270,315 | 34,700 | 27,489 | 118,453 | 2.201% | 0.224% | 5.593% |
| 2019 | 324,697,795 | 1,586,103 | 2,129,229 | 12,338,516 | 38,100 | 30,162 | 129,971 | 2.402% | 0.244% | 6.104% |

PWID = people who inject drugs; IDU = injection drug use; LB = lower bound; UB = upper bound; HCV = hepatitis C virus

^a^PWID population was estimated from a prevalence of IDU within the past 12 months of 1.8% (95% CI 0.9%-3.8%)^14^

**Table B4. Target estimates for serious bacterial infections hospitalization rates among active PWID in the US from 2015-2017.**

|  |  | **PWID Population^a^** | | | **Number of hospitalizations for bacterial infections from IDU^b^** | | | | | **Calculated hospitalization rate among active PWID** | | |
| --- | --- | --- | --- | --- | --- | --- | --- | --- | --- | --- | --- | --- |
| **Yr.** | **Total Pop. ≥18y** | **Estimate** | **LB** | **UB** | **Endo-carditis** | **Osteo-myelitis** | **CNS infections** | **SSTI** | **TOTAL** | **Est.** | **LB** | **UB** |
| 2015 | 247,789,111 | 1,015,935 | 867,262 | 1,164,609 | 3,471 | 4,482 | 1,480 | 71,736 | 81,168 | 8.0% | 7.0% | 9.4% |
| 2016 | 249,489,772 | 1,022,908 | 873,214 | 1,172,602 | 3,834 | 4,983 | 1,718 | 75,000 | 85,534 | 8.4% | 7.3% | 9.8% |
| 2017 | 252,070,495 | 1,033,489 | 882,247 | 1,184,731 | 5,098 | 5,938 | 2,176 | 80,855 | 94,068 | 9.1% | 7.9% | 10.7% |

PWID = people who inject drugs; IDU = injection drug use; LB = lower bound; UB = upper bound; CNS = central nervous system; SSTI = skin/soft tissue infections

^a^PWID population was estimated from a prevalence of IDU within the past 12 months of 0.41% (95% CI 0.35%-0.47%)^7^

**^b^**Includes IDU and IDU+MSM categories^8^

**Table B5. Annual fatal opioid overdose rate output for parameter sets meeting convergence criteria**

|  | **Lower**  **Bound** | **Upper**  **Bound** | **Estimate (99% CI)** | **Parameter set 1** | **Parameter set 2** | **Parameter set 3** |
| --- | --- | --- | --- | --- | --- | --- |
| **2015** | 0.15% | 0.14% | 0.15% | 0.15% | 0.15% | 0.15% |
| **2016** | 0.19% | 0.19% | 0.21% | 0.19% | 0.19% | 0.19% |
| **2017** | 0.23% | 0.22% | 0.25% | 0.23% | 0.24% | 0.24% |
| **2018** | 0.24% | 0.23% | 0.26% | 0.23% | 0.25% | 0.25% |
| **2019** | 0.27% | 0.25% | 0.29% | 0.28% | 0.29% | 0.29% |
| **Pearson's Chi-square:** | | | | 1.56E-05 | 2.65E-05 | 2.29E-05 |

**Figure B1. Internal validation of hospitalization rates for serious bacterial infections from 2015-2017.**


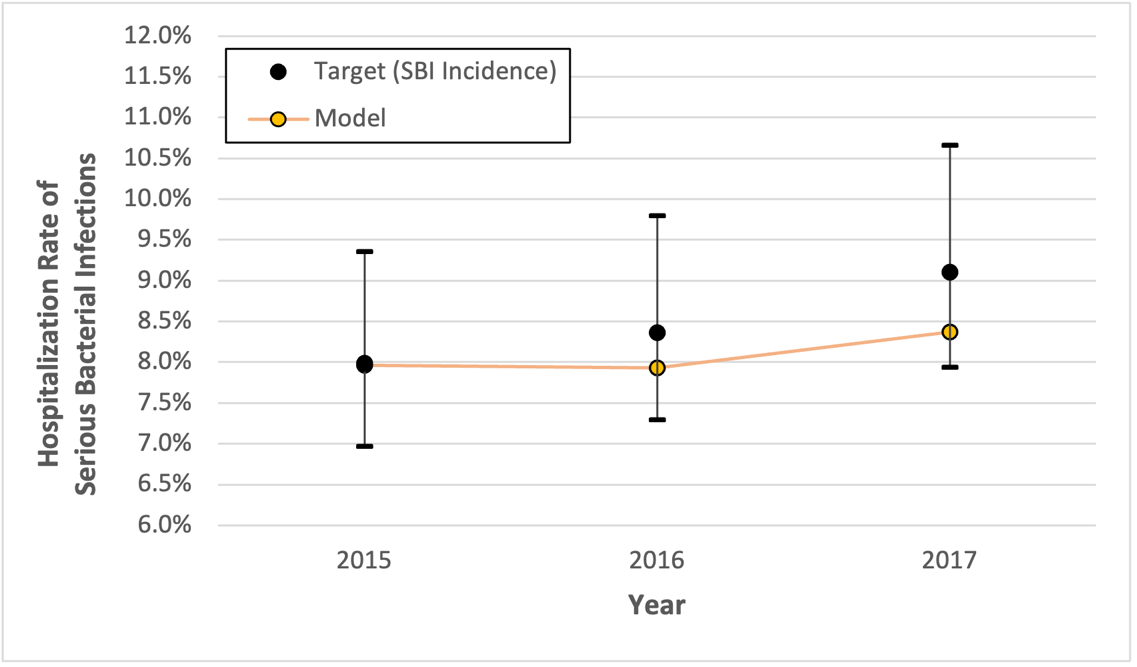


**Figure B2. Internal validation of HIV incidence from 2015-2019.**


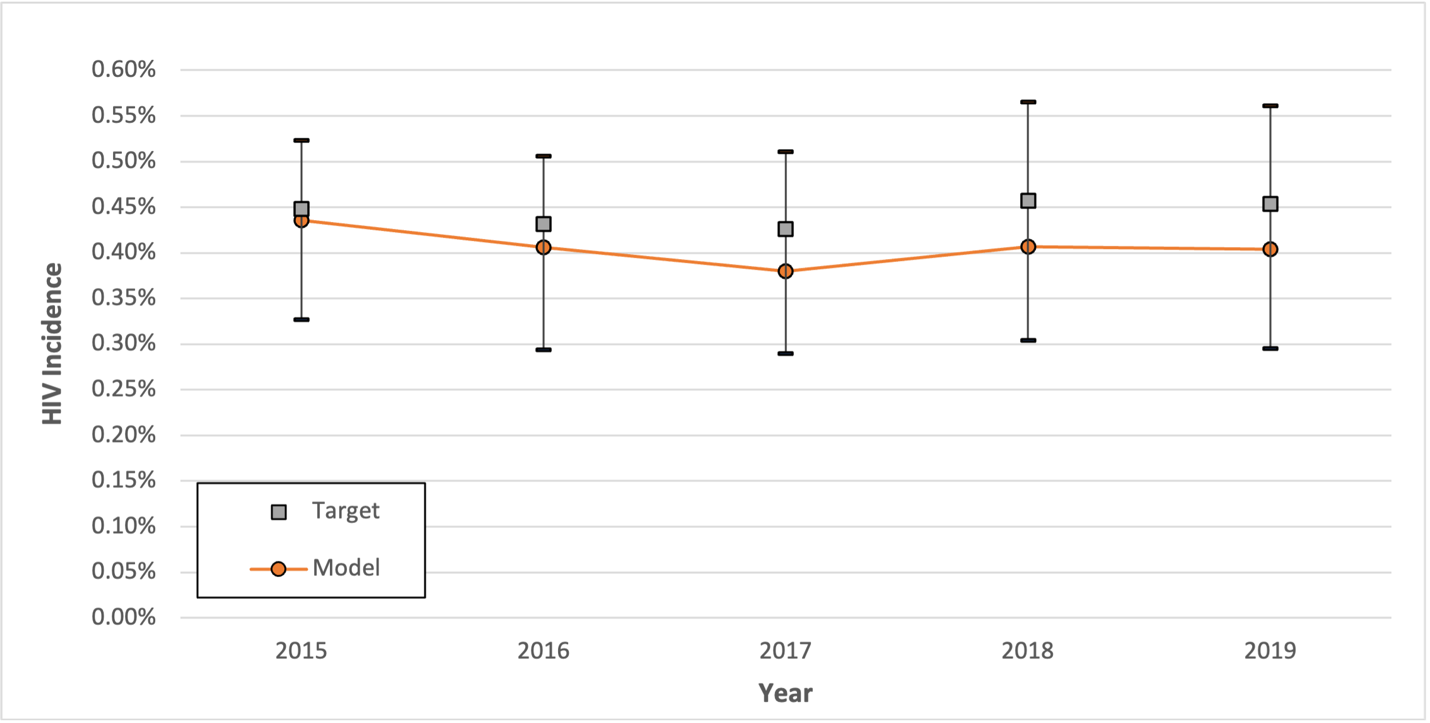


**Figure B3. Internal validation of HIV prevalence from 2015-2019.**

**
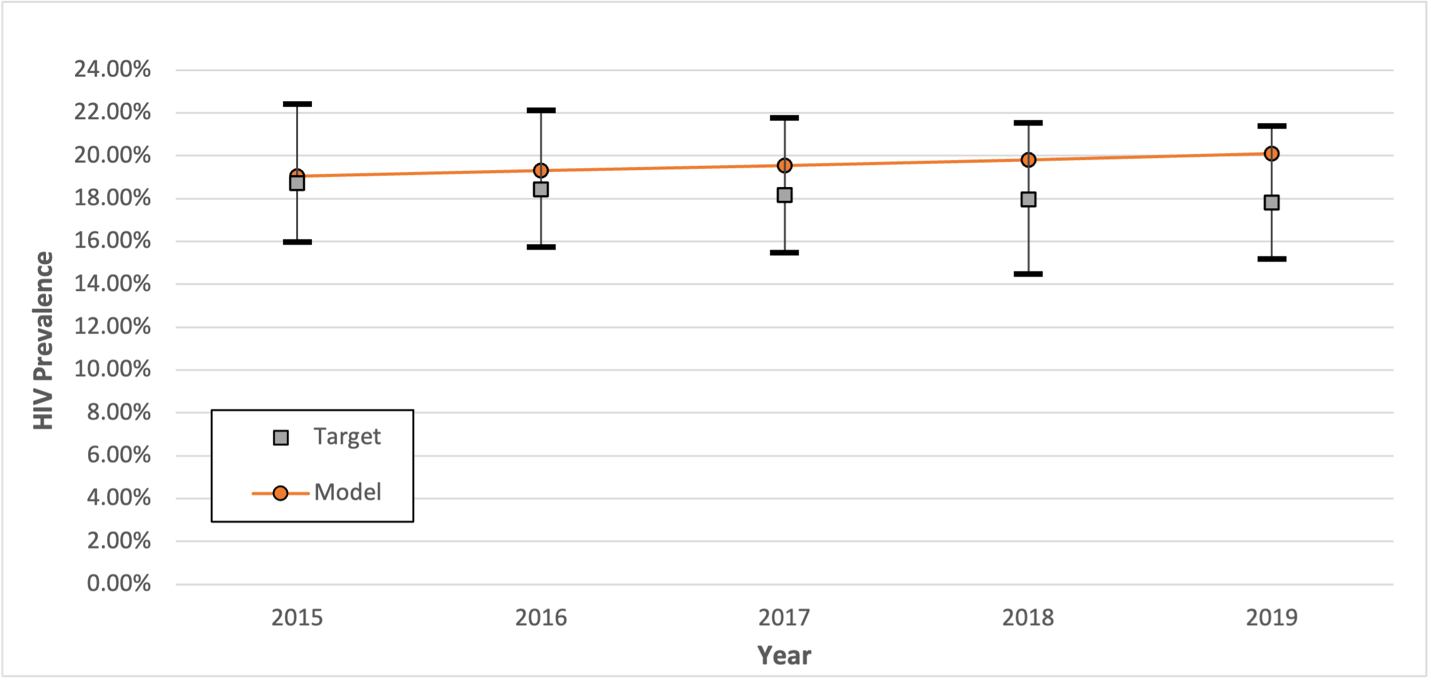
**

**Figure B4. Internal validation of HCV incidence from 2015-2019.**

**
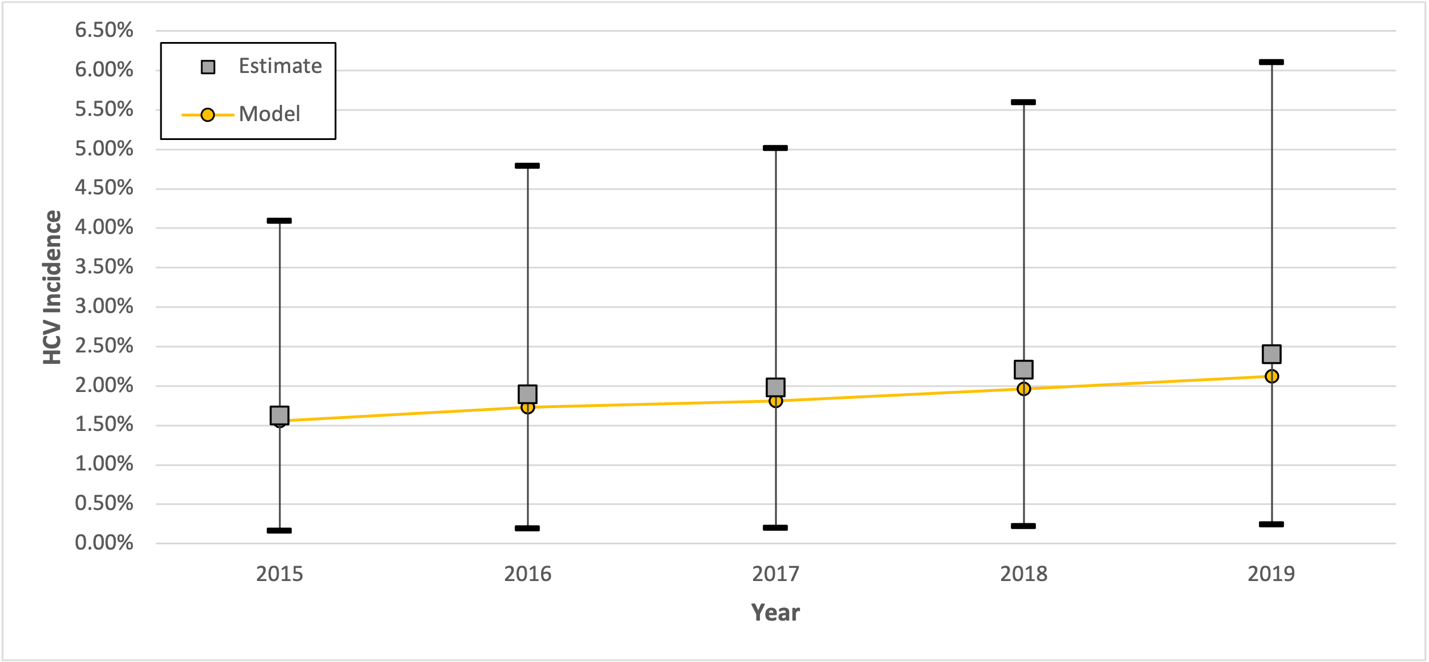
**

**Table B6. Reported use of opioids and injection from TEDS-A, 2015-2018**^19^

|  | **Heroin/Synthetic Opioids** | | **Natural/semi-synthetic opioids/methadone** | | **Total Opioids** | | |
| --- | --- | --- | --- | --- | --- | --- | --- |
| **Year** | **Reported injection** | **Reported use** | **Reported injection** | **Reported use** | **Calculated injection** | **Calculated use** | **Percent injection** |
| **2015** | 355,212 | 534,157 | 34,284 | 246,793 | 389,496 | 780,950 | 49.9% |
| **2016** | 374,358 | 562,792 | 34,315 | 249,220 | 408,673 | 812,012 | 50.3% |
| **2017** | 384,371 | 608,539 | 31,574 | 244,315 | 415,945 | 852,854 | 48.8% |
| **2018** | 347,020 | 576,569 | 31,534 | 232,349 | 378,554 | 808,918 | 46.8% |
|  |  |  |  |  |  | **Average** | **48.9%** |

**Table B7. Model-projected estimates of injection-related overdose deaths, 2015-2019**

| **Year** | **Average of model results for fatal opioid overdose rates** | **Estimated number of people who misuse opioids (95% CI)** | **Calculated number of opioid deaths (95% CI)** | **Calculated number of injection-related deaths (95% CI)^a^** |
| --- | --- | --- | --- | --- |
| **2015** | 0.1507% | 22,811,881  (21,696,430 - 23,927,332) | 34,370  (32,689 - 36,051) | 18,443  (17,541 - 19,345) |
| **2016** | 0.1893% | 21,582,291  (20,496,036 - 22,668,546) | 40,852  (38,796 - 42,908) | 21,921  (20,818 - 23,025) |
| **2017** | 0.2389% | 20,325,520  (19,212,701 - 21,438,339) | 48,567  (45,908 - 51,226) | 26,061  (24,634 - 27,488) |
| **2018** | 0.2437% | 19,081,554  (18,001,794 - 20,161,314) | 46,510  (43,878 - 49,142) | 24,957  (23,545 - 26,369) |
| **2019** | 0.2874% | 18,435,900  (17,170,618 - 19,701,182) | 52,989  (49,353 - 56,626) | 28,434  (26,483 - 30,385) |
| **Total** |  |  | 223,288  (210,610 – 235,952) | 119,816  **(**113,021 - 126,612**)** |

^a^ Calculated with data from TEDS-A that approximated 48.9% of opioid overdose deaths were injection-related and 91.2% of injection-related deaths are from opioids^14^

**References**

1. US Department of Health and Human Services, Substance Abuse and Mental Health Services Administration, Center for Behavioral Health Statistics and Quality. National Survey on Drug Use and Health (NSDUH), 2015-2020 [accessed 2022 Sep 12];Available from: https://www.datafiles.samhsa.gov/.

2. Barocas JA, White LF, Wang J, et al. Estimated Prevalence of Opioid Use Disorder in Massachusetts, 2011-2015: A Capture-Recapture Analysis. Am J Public Health 2018;108(12):1675–81.

3. Maruschak LM, Bronson J, Alper M. Alcohol and drug use and treatment reported by prisoners. (Rep. No. NCJ #252641). Washington, DC: United States Department of Justice, Bureau of Justice Statistics. Available from https://bjs.ojp.gov/sites/g/files/xyckuh236/files/media/document/adutrpspi16st.pdf.

4. Tsai J. Lifetime and 1-year prevalence of homelessness in the US population: results from the National Epidemiologic Survey on Alcohol and Related Conditions-III. J Public Health 2018;40(1):65–74.

5. McCarthy NL, Baggs J, See I, et al. Bacterial Infections Associated With Substance Use Disorders, Large Cohort of United States Hospitals, 2012–2017. Clin Infect Dis 2020;71(7):e37–44.

6. Kim J-H, Fine DR, Li L, et al. Disparities in United States hospitalizations for serious infections in patients with and without opioid use disorder: A nationwide observational study. PLoS Med 2020;17(8):e1003247.

7. Lansky A, Finlayson T, Johnson C, et al. Estimating the number of persons who inject drugs in the united states by meta-analysis to calculate national rates of HIV and hepatitis C virus infections. PLoS One 2014;9(5):e97596.

8. Centers for Disease Control and Prevention. Estimated HIV incidence and prevalence in the United States, 2015–2019. HIV Surveillance Supplemental Report 2021 [accessed 2021 Sep 4];26(1). Available from: https://www.cdc.gov/hiv/library/reports/hiv-surveillance.html.

9. Krebs E, Zang X, Enns B, et al. Ending the HIV Epidemic Among Persons Who Inject Drugs: A Cost-Effectiveness Analysis in Six US Cities. J Infect Dis 2020;222(Suppl 5):S301–11.

10. Long EF, Mandalia R, Mandalia S, Alistar SS, Beck EJ, Brandeau ML. Expanded HIV testing in low-prevalence, high-income countries: a cost-effectiveness analysis for the United Kingdom. PLoS One 2014;9(4):e95735.

11. Wu K, Tie Y, Dasgupta S, Beer L, Marcus R. Injection and Non-Injection Drug Use Among Adults with Diagnosed HIV in the United States, 2015-2018. AIDS Behav 2021;26(4):1026-1038.

12. Kabiri M, Jazwinski AB, Roberts MS, Schaefer AJ, Chhatwal J. The changing burden of hepatitis C virus infection in the United States: model-based predictions. Ann Intern Med 2014;161(3):170–80.

13. Centers for Disease Control and Prevention. Viral Hepatitis Surveillance 2019. U.S. Department of Health and Human Services; 2021 [accessed 2022 Oct 10]. Available from: https://www.cdc.gov/hepatitis/statistics/2019surveillance/index.htm

14. Bradley H, Hall E, Asher A, et al. Estimated number of people who inject drugs in the United States. Clin Infect Dis 2022;76(1):96-102.

15. Grebely J, Page K, Sacks-Davis R, et al. The effects of female sex, viral genotype, and IL28B genotype on spontaneous clearance of acute hepatitis C virus infection. Hepatology 2014;59(1):109–20.

16. Engels EA, Yanik EL, Wheeler W, et al. Cancer-Attributable Mortality Among People With Treated Human Immunodeficiency Virus Infection in North America. Clin Infect Dis 2017;65(4):636–43.

17. Hessol NA, Kalinowski A, Benning L, et al. Mortality among participants in the Multicenter AIDS Cohort Study and the Women’s Interagency HIV Study. Clin Infect Dis 2007;44(2):287–94.

18. HIV-CAUSAL Collaboration, Ray M, Logan R, et al. The effect of combined antiretroviral therapy on the overall mortality of HIV-infected individuals. AIDS 2010;24(1):123–37.

19. Hall EW, Rosenberg ES, Jones CM, Asher A, Valverde E, Bradley H. Estimated number of injection-involved drug overdose deaths, United States, 2000 - 2018. Drug Alcohol Depend 2022;234(109428):109428.
